# How Imaging Techniques Improve Ventricular Arrhythmia Ablation: A Multimodality-Based Approach

**DOI:** 10.3390/jcm12237420

**Published:** 2023-11-30

**Authors:** Antonio Gianluca Robles, Matevž Jan, Tine Prolič Kalinšek, Bor Antolič, Martin Rauber, Luka Klemen, Matjaž Šinkovec, Silvio Romano, Luigi Sciarra, Andrej Pernat

**Affiliations:** 1 Department of Life, Health and Environmental Sciences, University of L’Aquila, 67100 L’Aquila, Italy; silvio.romano@univaq.it (S.R.); luigi.sciarra@univaq.it (L.S.); 2 Department of Cardiology, “L. Bonomo” Hospital, 76123 Andria, Italy; 3 Department of Cardiology, University Medical Centre Ljubljana, 1000 Ljubljana, Slovenia; bor.antolic@kclj.si (B.A.); martin.rauber@kclj.si (M.R.); luka.klemen@kclj.si (L.K.); matjaz.sinkovec@kclj.si (M.Š.); andrej.pernat@kclj.si (A.P.); 4 Department of Cardiovascular Surgery, University Medical Centre Ljubljana, 1000 Ljubljana, Slovenia; matevz.jan@kclj.si (M.J.); tine.prolic.kalinsek@kclj.si (T.P.K.)

**Keywords:** ventricular tachycardia, cardiac magnetic resonance, cardiac computed tomography, nuclear techniques, intracardiac echography, electroanatomic mapping

## Abstract

Although implantable cardioverter defibrillators offer the best protection against sudden cardiac death, catheter ablation for ventricular arrhythmias (VAs) can modify or prevent this event from occurring. In order to achieve a successful ablation, the correct identification of the underlying arrhythmogenic substrate is mandatory to tailor the pre-procedural planning of an ablative procedure as appropriately as possible. We propose that several of the imaging modalities currently used could be merged, including echocardiography (also intracardiac), cardiac magnetic resonance, cardiac computed tomography, nuclear techniques, and electroanatomic mapping. The aim of this state-of-the-art review is to present the value of each modality, that is, its benefits and limitations, in the assessment of arrhythmogenic substrate. Moreover, VAs can be also idiopathic, and in this paper we will underline the role of these techniques in facilitating the ablative procedure. Finally, a hands-on workflow for approaching such a VA and future perspectives will be presented.

## 1. Introduction

In light of the latest guidelines and scientific data, there is increasing support for early ablative treatment for ventricular arrhythmias (VAs), including idiopathic premature ventricular complexes (PVCs) and/or non-sustained ventricular tachycardias (NSTVs), like right ventricular outflow tract (RVOT) VAs, especially in the case of PVC-induced cardiomyopathy. Importantly, there is also increasing support for the early ablation of ischemic VT [1,2,3].

Given that catheter ablation is currently considered the most effective non-pharmacological treatment in reducing the burden and recurrence of VAs, the ablation strategy as well as the effectiveness of the ablation itself are strongly related to the precise characterization of the determining substrate when it is present [4,5,6].

Generally speaking, when approaching the management of such a VA (e.g., PVC or VT), the most important thing is to determine the presence and the type of an underlying cardiomyopathy upon which the prognosis and treatment strategy depend.

Exceptions are polymorphic VTs related to inherited channelopathies (BrS, LQTS, SQTS, CPVT, ERS, etc.) or reversible acute events (ischemia, electrolytes imbalance, drugs, etc.).

In this presentation, we will focus on PVCs and monomorphic VTs that could be idiopathic or substrate related [3].

The latter are often simplistically subdivided into ischemic (IHD) or non-ischemic (NIHD). On the other hand, non-ischemic substrates are a heterogeneous group, taking into account dilated cardiomyopathy, hypertrophic cardiomyopathy, arrhythmogenic cardiomyopathy, cardiac sarcoidosis, valvular heart diseases, infiltrative diseases, congenital heart diseases, Chagas’ disease, and a newly recognized scar-related cardiomyopathy: arrhythmogenic mitral valve prolapse [3].

The aim of this paper is to examine the currently available imaging techniques with diagnostic and therapeutic value in VA ablation. In particular, we will appreciate their aid in recognition of the presence and extent of structural heart disease (SHD), arrhythmogenic substrate localization and characterization, and procedural planning. From a mere procedural point of view, some of them allow a reduction in the procedural use of fluoroscopy, improvement of ablative lesion quality through real-time monitoring of tip/tissue contact, and improvement of safety with early detection of complications. Additionally, available techniques substantially improve understanding anatomy relevant for ablation and can help rule out any structural anomalies in idiopathic VAs. The possibility of correctly evaluating the structure and ejection fraction as well as fibrosis—especially with CMR and CT—enables a fine characterization of the heart disease on which the prognosis of the patient with VAs depends. 

**Figure 1:**
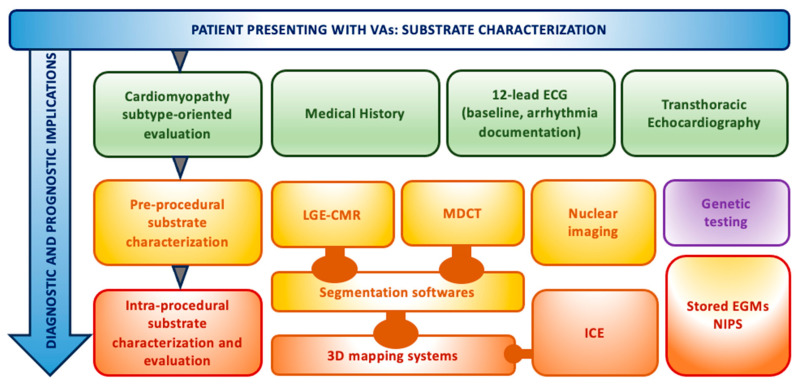
Schematic stepwise approach to substrate definition for VA ablation. The different techniques can be combined for each patient in a tailored approach in order to better evaluate the presence, localization, and extension of the substrate. Notably, data have diagnostic and prognostic implications. To this extent, genetic testing also deserves attention in this sophisticated algorithm.

The following techniques are primarily used: echocardiography (and, in particular, intracardiac echocardiography (ICE), cardiac magnetic resonance (CMR), cardiac computed tomography (CT), and three-dimensional (3D) mapping systems. They can be exploited differently and combined in a stepwise approach for the arrhythmic substrate definition (Figure 1).

## 2. Pre-Procedural Assessment

Before the examination of the available techniques, it is important to keep in mind a patient’s clinical and family history (presence of cardiomyopathy and/or SCD in first-degree relatives) and available ECG recordings (baseline 12-lead ECG and recordings during PVC or VT) in order to choose the most appropriate next step in planning the ablative procedure [3].

To this extent, the morphology of arrhythmia itself suggests the heart chamber of focus or at least in which heart chamber to start the mapping [3,7].

This, together with data on the underlying cardiomyopathy (if it is present), may orient the operator to start the mapping endocardially or epicardially. The ECG criteria suggesting epicardial origin of VAs are summarized in Table 1 [8,9], highlighting the importance of the ECG-derived clues regarding the possible substrate [10]. Indeed, ischemic forms have more frequently endocardial scar and VT exits, but non-ischemic ones have a variable and consistent epicardial and/or mid-wall substrate localization. And, as we will see later, substrate localization in the latter can be appropriately characterized with CMR and/or CT. 

An interesting and feasible approach in VAs exit prediction comes from the electrocardiographic imaging (ECGi) which integrates data from body surface mapping and 3D heart anatomy derived from CMR or CT [11,12,13,14,15].

However, ECG traces of the clinical arrhythmia are not always available. In these cases, baseline 12-lead ECG may suggest scar location in patients with signs of previous myocardial infarction (Q waves, poor R waves and/or persistent ST elevation suggesting the site of previous necrosis and ventricular aneurysm, respectively); conversely, anteroseptal or inferolateral epicardial arrhythmic substrates in patients with NIHD are, respectively, suggested by conduction disturbances or QRS abnormalities in lateral leads predominantly. Table 2 summarizes the possible non-ischemic substrate-related abnormalities of native QRS [9,16].

Otherwise, especially in NIHD patients, VT can be induced by using ICD for non-invasive programmed stimulation (NIPS) or by EP study before ablation in order to obtain clues about possible substrate localization. Notably, in patients without previous documentation of VT on the 12-lead ECG due to appropriate ICD treatment, clinical VT during the procedure may be recognized by comparing its cycle length and real-time EGMs morphology displayed on the programmer with the ICD-stored VT episodes [17,18].

ICD-stored clinical VT episodes may help with differentiating antero-septal vs. inferolateral and endocardial vs. epicardial VTs exits by evaluation of timing between far-field and near-field EGMs. In particular, timing ≤20–30 ms suggests antero-septal or endocardial exits; conversely, timing ≥60 ms suggests inferolateral or epicardial exits [19].

### 2.1. Transthoracic Echocardiography

After the 12-lead ECG, transthoracic echocardiography (TTE) is the first exam performed in order to evaluate cardiac morpho-functional parameters. They include heart chambers size measurement, evaluation of left ventricular ejection fraction (LVEF) and valves function. Of note, ultrasound allows the detection of intracardiac thrombi whose presence will address the procedure rescheduling to allow for an appropriate anticoagulation period and/or to direct epicardial mapping [5]. Of importance, thrombi identification may increase with the use of contrast, especially if the LV endocardium is not well visualized [20,21]. In the context of ischemic cardiopathy, TTE identifies areas of wall motion abnormalities, aneurysms and reduced wall thickness, which correlate with low-voltage areas during 3D mapping and the presence of VT critical isthmuses/exits. 

On the other hand, TTE is of less aid in the pre-procedural planning of VTs related to NIHD. Even if it allows the differentiation of subforms like hypertrophic phenotype vs. dilated phenotype, it does not allow accurate differentiation regarding the etiology and substrate localization. Indeed, in the hypertrophic phenotype, one can identify pure subsets of hypertrophic cardiomyopathy (genetically inherited forms), Fabry’s disease, amyloidosis, etc., whose correct identification is based on clinical, laboratory, genetic and, in some cases, cardiac nuclear essays [22,23].

The dilated phenotype subgroup encompasses pure dilated cardiomyopathy (genetically inherited forms in 40%) but also post-myocarditis DCM (6% of such patients), arrhythmogenic cardiomyopathy (especially in bi-ventricular/end-stage forms), advanced/terminal expression of valvular and corrected congenital heart diseases and cardiac sarcoidosis [24,25].

All these pathologies share the presence of fibrotic or fibro/fatty myocardial replacement with a variable degree of ventricular (LV or RV, or both) involvement.

Arrhythmogenic mitral valve prolapse is an emerging cause of VAs and sudden death. In this context, the presence of mitral annular disjunction (MAD), curling of the posterior leaflet and moderate-severe mitral regurgitation have been recognized as a marker of VAs risk, again, together with the presence and the extension of non-ischemic scars [26,27].

TTE helps with the detection of right heart failure that substantially increases procedure-related complications and, moreover, in case of severe left ventricle impairment (LVEF ≤25%—among the PAINESD risk score items), it is reasonable to consider prophylactic procedural hemodynamic support with extracorporeal membrane oxygenation (ECMO), especially in NIHDs in which a substrate modification approach based on LAVAs/LPs and/or pace mapping is less effective in comparison with IHD [28,29]. 

### 2.2. Cardiac Magnetic Resonance

CMR has a fundamental role in the pre-procedural assessment of patients with VAs: to diagnose or exclude the presence of a scar-related structural heart disease and, if present, to characterize it. From this point of view, it has a prognostic value, since the elimination of the arrhythmogenic focus alone represents the conclusion of the treatment in patients without SHD, and a substantial improvement, or even normalization, of the LVEF is often observed. Indeed, differently from NICM patients, PVC-induced cardiomyopathy patients usually have no myocardial late gadolinium enhancement (LGE) presence. These data enable differentiating the two forms of cardiopathy and predicting the chance of recovery at the same time [30].

On the contrary, in the presence of heart disease, the localization of the scar is particularly useful for planning the ablation procedure. Importantly, the initial mapping approach can be planned as endocardial, epicardial or both. In fact, it has been demonstrated that the use of CMR in pre-procedural planning improves the outcome of patients with IHD and NIHD by reducing VT recurrences and mortality and, at the same time, assessing the region of interest in advance reduces procedural time [31,32,33,34,35].

In this context, CMR is less important in ischemic subsets, since in most cases, the mapping and ablation are limited to the endocardial layer where the region of interest can often be suggested by careful evaluation of the 12-lead ECG and/or the transthoracic ultrasound. However, its usefulness remains undisputed in identifying a minority of ischemic patients with transmural scars who can benefit from an endo-epicardial ablative strategy. On the other hand, CMR has higher accuracy than TTE in identifying myocardial thrombi [36,37,38]. Surely, the role of CMR is essential in the localization of scar in NIHD forms. In fact, CMR has been shown to be superior to endocardial unipolar EAM for the detection of mid-wall arrhythmogenic substrates [31,33,35].

The last European guidelines for the management of cardiomyopathies distinguish NICM into different forms (HCM, DCM, NDLVC, ARVC, RCM, etc.) according to their phenotypes [39]. Roughly, DCMs can be divided into two groups based on the scar distribution: forms with basal antero-septal scars and those with scars localized in the basal inferolateral segments [16]. The former generally show a mid-wall scar and are typically genetically determined DCM (LMNA A/C or titin mutations), and less commonly, they are due to prior myocarditis [40]. The latter are generally consequences of previous myocarditis and only in a minority of cases are they due to genetic mutations (such as phospholamban, which is responsible for fibro-fatty replacement). Another pattern that strongly suggesting inherited DCM is the “ring-like” scar due to desmoplakin/filamin C mutations, which is associated with a high risk of polymorphic VAs and SCD [41].

To date, the gold standard for scar detection is LGE, but we should take into account that there is also an alternative promising technique to improve myocardium characterization: T1 mapping. It allows the evaluation of extracellular volume fraction that fully quantifies myocardial fibrosis and also iron, glycosphingolipid or amyloid deposits [42].

Although the CMR is mandatory for all the highlighted reasons, it is not without limits. First, its 1–2 mm spatial resolution makes evaluation less precise in thin walls such as RV and in the presence of numerous irregular contractions (e.g., high PVC burden). Second, there is no standardized technique for the assessment of tissue heterogeneity (which is essential for the distinction between dense scar and border zones): two techniques exist, but they are not histologically validated. Furthermore, the CMR is limited by artifacts caused by cardiac implantable electronical devices (CIED). For this reason, it is wise to perform the exam before ICD implantation (preemptive CMR). Of note, wideband LGE-CMR partially overcomes device artifacts [29,43].

### 2.3. Cardiac Computed Tomography

The advantage of multidetector cardiac CT (MDCT) over LGE-CMR is the higher spatial resolution that is in the sub-millimeter range. Various MDCT characteristics indicative of scarring have been described, including a degree of wall thinning with a cutoff wall thickness of <5 mm, hypoattenuation and delayed iodine enhancement [44,45,46].

More recently, thanks to the higher spatial resolution of MDCT, thicker parts of tissue separating areas of thinning have been described within the myocardial scar harboring the majority of VT target sites in ischemic patients [47]. Hence, MDCT imaging has been found to be beneficial as an alternative to CMR in the delineation of myocardial scarring especially in the presence of contraindications for CMR (i.e., CIED). Although assessment of wall thickness in MDCT imaging has been used successfully in patients with prior myocardial infarctions, the ability to identify scarring based on wall thickness alone has been less successful in patients with NICM and might require a different approach [45,48,49].

MDCT is the imaging technique of choice for pre-procedural imaging of the coronary arteries, the coronary veins, and the phrenic nerves. The ability to image the coronary arteries in conjunction with the ability to image epicardial fat thickness has been especially valuable for epicardial ablation procedures to enhance safety and to quantify epicardial fat thickness covering potential epicardial VT origin sites [50,51]. On the other hand, MDCT is inferior to MRI in myocardial tissue characterization, and it needs significant radio exposure to achieve a good image quality [52]. These, together with artifacts sometimes produced by CIED leads and high heart rate and/or PVC burden, represent important CT limitations.

Photon-counting computed tomography (PCCT) is a new advanced imaging technique that is expected to transform the standard clinical use of computed tomography (CT) imaging. Compared to conventional CT technology, PCCT offers the advantages of improved spatial and contrast resolution, reduction in image noise and artifacts, reduced radiation exposure, and multi-energy/multi-parametric imaging based on the atomic properties of tissues with the consequent possibility of using different contrast agents and improving quantitative imaging [53].

### 2.4. Segmentation Software

To date, there are two available types of segmentation software: inHeart and ADAS 3D. Both operate with imaging acquisition through CT or CMR and enable processed images to be imported in the major three-dimensional (3D) electro-anatomical mapping (EAM) systems available (see below). Both allow delineation not only of the wall thickness and scar localization but also an accurate delineation of coronary arteries, venous system, phrenic nerves and CIED leads. In particular, the possibility to import these structures into the 3D EAM systems reduces the necessity for fluoroscopy or coronary angiography. Both software also allow manual or automatic generation of scar maps based on LGE or late iodine enhancement that can also be imported into the 3D EAM systems. These scar maps depict “border-zone corridors” of viable myocardium inside dense fibrosis that correlate with arrhythmogenic isthmuses and thus are ablation targets. In addition, there are some studies supporting the use of inHeart and ADAS 3D for the pre-procedural planning. Those studies demonstrate their effect on reduction in procedural time and arrhythmic recurrences [43,54,55,56,57].

These software have some limitations. Firstly, the accuracy and usability of processed images with arrhythmogenic substrate depiction heavily depend on the quality of CT or MRI-acquired images. Secondly, the accuracy of merging into the 3D EAM can vary with variable anatomy (i.e., chamber volume and heart orientation), which can have an impact on otherwise standardized merging protocols. Thirdly, a possibly long time interval between image acquisition and its use for segmentation can have an impact on anatomical accuracy at the actual time of the procedure [43,58].

**Figure 2:**
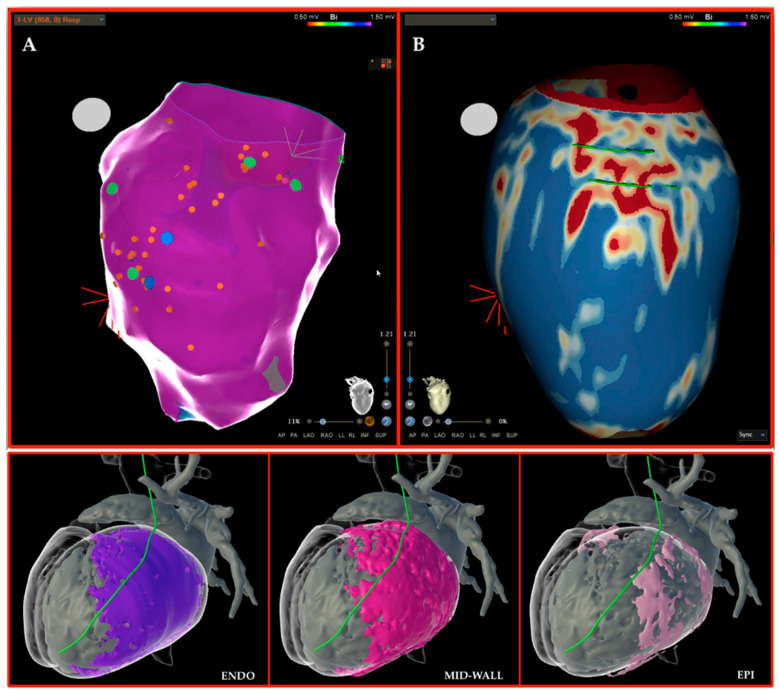
Examples of segmentation software applications. **Upper Panel**. (**A**) shows a case of an NICM patient and recurrent VT in which the endocardial substrate high-density mapping does not show low-voltage areas, except for a few abnormal ventricular EGM signals (green, blue and orange tags). Conversely, in (**B**), the ADAS 3D anatomical reconstruction of the LV epicardium obtained with processing LGE-CMR imaging is shown and, in particular, it is evident that scar localization and putative channels (green lines) are at the inferolateral basal wall. On the basis of this, the patient was submitted to an epicardial procedure. **Lower Panel**. A case of another patient with a genetically determined NICM and a transmural “ring-like” scar mostly affecting the lateral wall. From left to right, endocardial, mid-wall and epicardial layers are displayed, and it is clear that the major extension of the scar is in the endocardial/mid-wall layers rather than the epicardial one. In this patient, an inHeart reconstruction of the anatomical structures and the scar was made with processing CT-scan and late iodine enhancement imaging. The left phrenic nerve is shown in green.

Figure 2 shows two examples of clinical applications of the mentioned segmentation software.

### 2.5. Nuclear Imaging

Positron emission tomography (PET) and single-photon emission computed tomography (SPECT) may have a place in pre-procedural planning. A correlation between scars detected by PET/CT and EAM was demonstrated. The same is true for SPECT, however, with a lower correlation [59,60]. Interestingly, PET may identify a viable myocardium in dense scars, suggesting a potential ablative target. Importantly, it may also suggest the inflammatory etiology of the scar, such as sarcoidosis [61]. Instead, SPECT may detect sympathetic denervated myocardium, which is a sensitive marker of arrhythmic development [62,63,64,65].

These metabolic/functional imaging techniques have the limitation of low spatial resolution and thus are not practically useful for segmentation, processing and the accurate identification of mapping and ablation targets, as was demonstrated for CT and CMR. 

## 3. Intra-Procedural Assessment

### 3.1. Intracardiac Echography

Given the known effect of X-rays on the biological tissue of both patients and medical staff, the American College of Cardiology recommends the adoption of the “ALARA” principle: radiation doses should be “As Low as Reasonably Achievable” [66].

**Figure 3:**
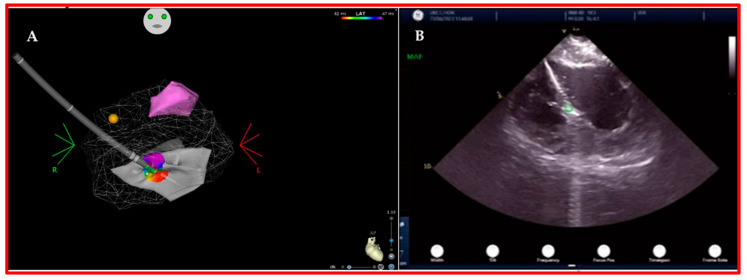
Left postero-medial papillary muscle (PM) PVC mapping and ablation. Panel (**A**) shows papillary muscles’ 3D anatomy reconstructed with ICE (antero-lateral PM is pink colored; postero-medial PM is in gray, and a point-by-point activation map is displayed) inside the mesh anatomy of the left ventricular endocardium. Yellow dot represents distal HIS bundle. Panel (**B**) shows a real-time ICE view of the mapping/ablation catheter tip (green circle) in contact with the tip of the postero-medial PM. In particular, ICE not only gives feedback in regard to the position and stability of the catheter tip in relation to the PM but also helps in recognizing the particular anatomy of the PM that sometimes has more than only one head.

The use of ICE seems to reduce and possibly eliminate the need for X-ray fluoroscopy by allowing the real-time imaging of catheters during mapping and ablation. Additionally, ICE enables the monitoring of possible procedural complications (especially pericardial effusion, steam pops, thrombi, air embolism and various causes for intra-procedural hypotension) and the direct imaging of anatomical variations and intracavitary structures (i.e., papillary muscles, false chordae and moderator band) (Figure 3) that cannot be adequately imaged with the 3D EAM systems [67,68]. Previous studies showed that a completely zero-fluoro approach to catheter ablation is feasible and safe in most types of VAs even when SHD is present [69,70].

As anticipated, there are other advantages of ICE during the VAs mapping and ablation procedure given by the early detection of complications. In particular, with ICE, we can recognize an impending steam pop by a sudden whitening of the tissue, thus timely stopping RF erogation. These data are of particular value when ablating in the ventricles (especially in the thicker left one) because we cannot rely on standardized parameters (i.e., Ablation Index or Lesion Size Index) that are surrogates of lesion goodness and size. To this extent, ICE may allow tailoring RF erogation point by point, especially when a deep substrate is suspected [24,71].

Furthermore, ICE gives real-time feedback on tissue/tip contact and stability during mapping and ablation, especially, as already stated, for intracavitary structures but also RV free-wall, perivalvular regions and the postero-superior process of the left ventricle [67]. Curiously, this last area may be the site of transseptal access from the RA to the LV in case of the so-called “locked LV” (a condition in which LV cannot be accessed antegradely nor retrogradely because of both the mechanical mitral and aortic valve) [72]. It is implicit that this kind of puncture may be performed only with ICE guidance, which obviates the need for cardiac surgery. To some extent, ICE permits the visualization of the location and extension of myocardial scars, which might be particularly of value when mapping the non-ischemic substrate. The enhanced echogenicity seen with ICE was found to correlate with low-voltage areas identified with 3D EAM [73]. Indeed, the potential identification of mid-wall and/or epicardial scars with ICE may reveal cases which deserve epicardial mapping. Importantly, ICE improves the monitoring of tissue contact of multipolar catheters (lacking contact-force sensors), thus avoiding possible false low-voltage detection caused by poor contact [29,74].

With primarily using a 3D EAM system and ICE, there seem to be two major limitations where the use of fluoroscopy cannot be avoided: (1) epicardial mapping and ablation where there is the need to visualize wires, long sheaths and catheters in the epicardial space; (2) application of hemodynamic support devices; and (3) the ablation of VAs originating in the LV summit where the visualization of coronary anatomy is necessary to avoid RF delivery close or on top of a large caliber coronary artery. Sometimes, the last issue can be partially overcome when mapping close to the ostial or very proximal part of the coronary vessel (i.e., left main/proximal left anterior descending/proximal left circumflex) where anatomy recognition and depiction by the aid of a SOUNDSTAR^®^ probe and CARTOSOUND^®^ module is helpful [67]. Also, the use of automated ICE and 3D EAM image integration with CARTOSOUND^®^ is valuable in the treatment of idiopathic and structural Vas, because it allows fast reconstruction of the anatomy of the outflow tracts and their related structures (i.e., pulmonary valve leaflets, sinus of Valsalva, aorto-mitral continuity, etc.) but also a quick depiction of the endocardial surface of the ventricles, allowing appreciation of their entire volume and identification of aneurysms when they are present. 

Finally, ICE is still underused given its prohibitive cost.

### 3.2. Three-Dimensional (3D)-Mapping Systems 

Three-Dimensional (3D) EAM systems definitely revolutionized the EP field since their adoption into clinical practice, which was now more than twenty years ago. There are three main EAM systems available: CARTO^®^ (Biosense Webster, Diamond Bar, CA, USA), EnSite^®^ (Abbott, St Paul, MN, USA), and RHYTHMIA HDx^®^ (Boston Scientific, Marlborough, MA, USA).

**Figure 4:**
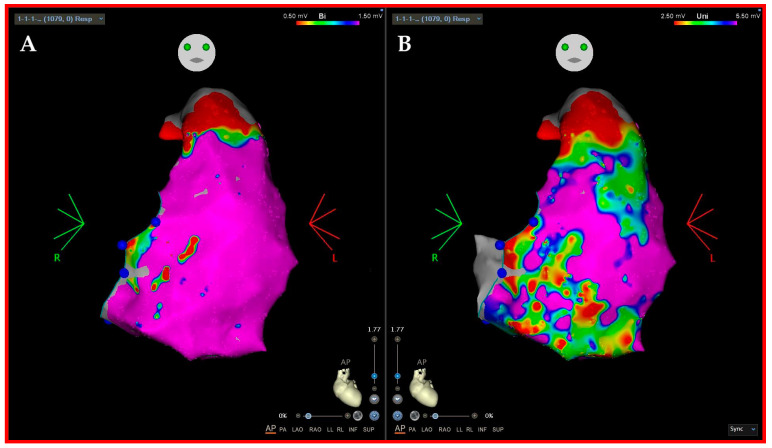
Arrhythmogenic Right Ventricular Cardiomyopathy (ARVC). Panel (**A**) shows endocardial bipolar voltage mapping of the RV (scar ≤0.5 mV; border zones 0.5–1.5 mV; healthy tissue >1.5 m). The RV endocardium appears more or less healthy with normal bipolar voltage represented by purple color. Panel (**B**) shows endocardial unipolar voltage mapping of the RV (scar ≤ 2.5 mV; border zones 2.5–5.5 mV; healthy tissue > 5.5 m). A diffuse low-voltage area on the anterior RVOT and on the inferolateral wall can be recognized (the so-called “displasia triangle”), suggesting a fibro-fatty replacement of the epi/mid-wall myocardial layer. This is a typical pattern seen in ARVC where pathological fibro-fatty replacement begins on the epicardial RV layer; then, it spreads through the mid-wall and only in later stages does it involve the endocardium.

Specifically, in the management of Vas, the 3D EAM systems are essential in fluoroscopy reduction; however, their key role is in substrate characterization and understanding the VT circuit in structural heart disease. In this regard, they can be used in conjunction with preprocedural imaging methods, as mentioned earlier. Importantly, CMR has limited spatial resolution (1–2 mm) and thus a limited power regarding the characterization of thin walls like those of the RV, where 3D EAM systems alone can offer insight into the characteristics of the arrhythmogenic substrate [5,75] (Figure 4). 

There are different strategies to approach the invasive mapping of monomorphic or scar-related VT. Originally, they were based on entrainment mapping, activation mapping and pace mapping. We can perform these strategies with the aid of multipolar mapping catheters and conventional fluoroscopic support. Alternatively, use of the 3D EAM systems provides propagation maps during VT, pace-mapping maps, pattern-matching algorithms and various advanced mapping tools [76]. Of note, less than one-third of VTs are hemodynamically stable and mappable or inducible in the EP lab. Consequently, various techniques of mapping the arrhythmogenic substrate in sinus (or paced) rhythm were developed in leading to the substrate modification method [77].

This is conceptually equivalent to the scar/aneurysm resection originally performed by cardiac surgeons to treat ischemic VTs [78,79]. In the concept of substrate modification, several mapping approaches are available, which are based on tagging and targeting different kinds of abnormal electrical signals found in diseased myocardium summarized by the term LAVA (local abnormal ventricular activity). This term encompasses fractionated, double, long (>80 ms) and late potentials (≥20 ms after the end of surface QRS) [80,81].

All identify viable myocardial bundles inside the scar and thus represent arrhythmogenic slow conducting channel surrogates with various sensitivity and specificity [82]. These signals may be detected in sinus and stable paced ventricular rhythm, allowing the creation of Isochronal Late Activation Maps (ILAMs) that help identify specific areas of conduction deceleration related to VT-generating slow-conducting channels. An important upgrade to the substrate-mapping method is Decrement-Evoked Potential (DeEP) mapping that is performed during programmed ventricular stimulation. It provides superior sensitivity in functional substrate characterization with the aim of unmasking abnormal deceleration of electrical conduction through the diseased myocardium [83,84].

Overall, VTs circuits simplistically need channels or isthmuses (slow conduction zones traversed by the wavefront of electrical activation during diastole), boundaries of conduction block (that may be fixed and due to fibrosis or also functional) and healthy tissue (represented by the rest of the ventricular myocardium activated during systole) [85]. Electro-anatomical voltage mapping with standardized parameters is the cornerstone for invasive scar detection and delineation [29]. During endocardial bipolar mapping, a dense scar is depicted by voltage ≤0.5 mV, border zones have voltages between 0.5 and 1.5 mV, and healthy tissue voltages ≥1.5 mV. The pathological electrical signals (LAVA, DeEP) are mostly, but not exclusively, found in areas with voltage <1.5 mV. For epicardial bipolar mapping, the thresholds differentiating dense scars from border zones and border zones from normal tissue are ≤0.5 mV and ≥1 mV, respectively. On the other hand, the unipolar endocardial mapping may suggest the transmural extension of the scar. In particular, voltages <5.5 mV or <8.3 mV are associated with deep (intramural, epicardial) scar in the RV or LV, respectively. Unipolar voltage mapping may be informative in cases where no abnormal electrical signals and/or no slow conduction zones are found with endocardial bipolar mapping [86] (Figure 4). This concept has added value in cases without pre-procedural or non-diagnostic MDCT or CMR, and it invariably has implications to ablative strategy and in the identification of concealed cardiomyopathy. It should be reiterated how energy choice and settings depend on substrate localization that could be targeted with different methods: RF (unipolar, sequential unipolar or bipolar; powered with or without half-saline or dextrose cooling solutions), cryo, PFA, RF-needle and stereotaxis radiotherapy. 

To summarize, 3D EAM systems allow substrate characterization and modification by a “probabilistic” approach targeting abnormal signals that reduces VTs recurrences compared with a “deterministic” approach based only on clinical VT/limited ablation in both ICM and NICM patients [82,87,88].

Last but not least, the amplitude of recorded electrograms depends on several technical issues, including but not limited to the size of mapping electrodes, interelectrode spacing, the orientation of the mapping catheter with respect to wavefront propagation, and the adequacy of catheter/tissue contact [74,89].

These mapping issues may be overcome by high-density omnipolar mapping catheters (HD Grid™ (Abbott, St Paul, MN, USA) and Optrell™ (Biosense Webster, Diamond Bar, CA, USA)) not affected by wavefront direction and, in the future, novel contact force-sensing ablation catheters equipped with microelectrodes may combine the advantages of high-resolution mapping with contact force-sensing capability [90,91,92].

## 4. Our Experience and Workflow

In light of all the aforementioned tools available, the starting point when approaching VAs is the clinical history of the patient. In a consistent number of cases, the presence of structural heart disease is known, and thus, the localization of the possible VT substrate can be anticipated. When unknown, first-line exams like 12-lead ECG (in baseline or during arrhythmia) and TTE give fundamental clues on the underlying cardiomyopathy when present. Often, ECG/TTE techniques are not sensitive enough to address scar/substrate localization, and it is recommended to perform LGE-CMR or, alternatively, MDCT, or rarely nuclear imaging. The evaluation of ICD-stored EGMs and NIPS can also be part of the pre-procedural assessment. Next, with all the accumulated data, the procedure can be planned. Its planning includes choice of access (endocardial vs. epicardial; transeptal vs. retroaortic) to the chamber of interest and ablation strategy. For example, for structural heart disease-related VTs, we usually use a transseptal approach to access the left ventricle with the aim of reducing or abolishing fluoro exposure, as previously described [69,93].

In general, we limit the retroaortic approach to the mapping and ablation of LV idiopathic PVCs/VTs originating in the LV outflow tract or aortic root and papillary muscle PVCs, especially those originating from the postero-medial one. CartoSound is routinely used for the 3D anatomy reconstruction of this structures, which also allows delineation of the proximal course of coronary arteries, which sometimes enables avoiding angiography when performing ablation in the vicinity of coronary arteries. A direct epicardial approach is performed in a first procedure when a clear epicardial substrate is known by pre-procedural imaging assessment or in a repeat procedure when pre-procedural assessment is not available, and electro-anatomical voltage and activation mapping and 12-lead ECG QRS morphology all point toward an epicardial site of origin. Epicardial access is obtained with percutaneous puncture, subxiphoid surgical window creation or rarely with left lateral thoracotomy in patients after previous bypass surgery or extensive pericardial adhesions. Choice of epicardial access is based on operator preference and patient characteristics (i.e., pericardial adhesions). A detailed MDCT, performed before the procedure, may in some cases reveal a close relationship between the epicardial substrate and a viable coronary venous branch that may allow epicardial mapping and ablation avoiding the pericardial access. Moreover, the use of fluoroscopy can be further reduced in epicardial procedures by merging the segmented anatomy of coronary arteries with the electro-anatomical maps. Finally, pre-procedural imaging may reveal a deep intramural septal substrate that may require bipolar ablation.

The procedural workflow is as follows:

1. A detailed substrate high-density mapping in sinus or paced rhythm (voltage, LAVA, LPs, DeEP mapping) performed by a multipolar-mapping catheter;
2. Induction of VT;
3. Pace mapping to find the site of interest according to the clinical or induced VT if it is not re-inducible or not hemodynamically tolerated;
4. In case of hemodynamically tolerated VT, we perform activation mapping to define the reentry circuit with areas of slow conduction coupled with entrainment mapping;
5. Ablation of LAVA/LP/DeEP at the site of interest in non-inducible or untolerated VTs or the slow conducting critical isthmus in tolerated VTs;
6. Complete substrate modification (elimination of all LAVA/LP/DeEP);
7. Re-mapping the ablated areas with a multipolar high-density mapping catheter;
8. Testing for final VT non-inducibility with programmed ventricular stimulation with up to four extrastimuli from two different sites with one of those close to the ablated low-voltage area. Isoproterenol administration during programmed ventricular stimulation depends on patient characteristics and operator’s preference.

## 5. Future Perspectives and Conclusions

In this narrative review, we have recounted the available imaging tools that increase ablation success and safety in the treatment of patients with VAs. The safety aspect applies not only to the patient but also to the physician and the EP lab staff by reducing or zeroing fluoro exposure. We think this concept is not trivial. 

Each technique has its advantages and limitations, and therefore, their combined use must be chosen based on the patient using a tailored approach that simultaneously takes into account the clinical implications and cost-effectiveness of each. If on the one hand, we have ECG and TTE as easy-to-use and low-cost techniques, the use of 3D-mapping systems is essential, and integration with CMR and CT data limited by reduced accessibility is becoming increasingly necessary. Furthermore, the processing of CMR and CT images to be imported into mapping systems requires professional technicians, further increasing time and costs. ICE has limited 3D-mapping systems integration possibilities and, once again, it is still underused for its high cost. Table 3 provides a synopsis of the pros and cons of some of the most important described imaging techniques.

The valuable information we can derive from careful evaluation of the ECG, cardiac ultrasound, second-line imaging methods (CMR/CT) and EAM techniques may simplify and, at the same time, improve procedure outcome. For sure, all of this will contribute to the consideration of catheter ablation as first-line therapy for Vas. For this purpose, further studies are needed, and new evidence from new techniques will be derived. In this context, we expect in the future more powerful and accurate CMR—also in real time—and CT imaging, epicardial space endoscopy, software for their integration with EAMs, implementation of 3D and 4D ICE, and new EAM techniques, both in sinus/paced rhythm or during VT like the novel VEDUM mapping (a color-coded mapping based on abnormal ventricular EGMs duration representing slow conduction areas) [94,95,96,97].

Finally, the blooming of artificial intelligence may bring us novelties about the automated localization of VT exit sites. A first experimental artificial non-invasive computational deep learning platform to localize VT exit sites from surface ECGs and device-stored EGMs has been developed, thus giving shape to this concept, which does not seem so far away [98].

## Figures and Tables

**Table 1:** The 12-lead ECG characteristics suggesting an epicardial origin of ventricular arrhythmias.

Pseudo-delta wave ≥34 ms in the precordial leads
Intrinsicoid deflection to R-wave peak in V2 ≥85 msec
Shortest RS duration ≥121 ms in any precordial lead
Maximum deflection index (MDI) ≥55 msec
Q wave in D1

**Table 2:** The 12-lead ECG characteristics of native QRS suggesting a non-ischemic substrate.

**Characteristics suggesting an antero-septal scar:**
AV-block
Left bundle branch block
Wide QRS
**Characteristics suggesting an inferolateral scar:**
Low QRS voltages in limb leads
No Q waves in inferior leads
QRS fragmentation in lateral leads
S/R ratio ≥0.25 in V6
r in V1 and s in V6 ≥0.15 mV

**Table 3:** Synopsis of pros and cons of some of the most important described imaging techniques. ECG, TTE, nuclear imaging and 3D-mapping systems are not included in this table for different reasons: (1) ECG and TTE are low-cost, essential, 1st-line exams; (2) conversely, nuclear imaging is uncommonly used because of its very strict indications and low spatial resolution; (3) 3D-mapping systems use is also essential in VAs ablation procedures. Therefore, attention was deliberately focused on those techniques whose costs and accessibility may still represent a limit in their use.

	CMR	MDCT	ICE
Radiation exposure	No	Yes	No
Scan duration	several minutes	few seconds	variable/real time
Planning benefits	pre-procedural	pre-procedural	intra-procedural
Fibrosis identification	++	+/-	+/-
Calcium identification	-	++	+/-
Thrombus identification	++	+	++
Coronary visualization/depiction	+/-	++	ostia and proximal tracts only
Fat identification	+	++	-
CIED generator artifacts	+++	+	-
CIED leads artifacts	+	+++	+
Integration with 3D-mapping systems	Yes	Yes	SOUNDSTAR/CARTOSOUND^®^ only
Segmentation software elaboration	Yes	Yes	No
Intracavitary structures visualization	Yes	Yes	Yes, real time

## Data Availability

The data that support the findings of this article are available from the corresponding author, [M.J.], upon reasonable request.

## Abbreviations

3D	Three-Dimensional
4D	Four-Dimensional
ARVC	Arrhythmogenic Right Ventricular Cardiomyopathy
BrS	Brugada Syndrome
CIED	Cardiac Implantable Electronic Device
CMR	Cardiac Magnetic Resonance
CPVT	Catecholaminergic Polymorphic Ventricular Tachycardia
CT	Computed Tomography
DCM	Dilated Cardiomyopathy
DeEP	Delayed Evoked Potential
EAM	Electro-Anatomical Mapping
ECG	Electrocardiogram
ECGi	Electrocardiographic Imaging
ECMO	Extra-Corporeal Membrane Oxygenation
EGM	Intracavitary Electrogram
ERS	Early Repolarization Syndrome
HCM	Hypertrophic Cardiomyopathy
ICD	Implantable Cardioverter Defibrillator
ICE	Intracardiac Echography
ICM	Ischemic Cardiomyopathy
IHD	Ischemic Heart Disease
ILAM	Isochronal Latest Activation Mapping
LAVA	Local Abnormal Ventricular Activity
LGE-CMR	Late Gadolinium Enhancement Cardiac Magnetic Resonance
LP	Late Potential
LQTS	Long QT Syndrome
LV	Left Ventricle
LVOT	Left Ventricular Outflow Tract
MAD	Mitral Annular Disjunction
MDCT	Multi-Detector Computed Tomography
NDLVC	Non-Dilated Left Ventricular Cardiomyopathy
NICM	Non-Ischemic Cardiomyopathy
NIHD	Non-Ischemic Heart Disease
NIPS	Non-Invasive Programmed Stimulation
NSVT	Non-Sustained Ventricular Tachycardia
PCCT	Photon-Counting Computed Tomography
PET	Positron Emission Tomography
PFA	Pulsed-Field Ablation
PM	Papillary Muscle
PVC	Premature Ventricular Complex
RCM	Restrictive Cardiomyopathy
RF	Radiofrequency
RV	Right Ventricle
RVOT	Right Ventricular Outflow Tract
SCD	Sudden Cardiac Death
SHD	Structural Heart Disease
SPECT	Single-Photon Emission Computed Tomography
SQTS	Short QT Syndrome
TEE	Transesophageal Echocardiography
TTE	Transthoracic Echocardiography
VA	Ventricular Arrhythmia
VT	Ventricular Tachycardia
